# Clinicians’ Experience of Remote Assessment of Autism Spectrum Disorder Within the Barnet CAMHS Service

**DOI:** 10.1192/bjo.2022.384

**Published:** 2022-06-20

**Authors:** Simona Constantinescu, Jaya Gupta, Itit Arora Fedyushkin, Adriana Fernandez-Chirre

**Affiliations:** 1 Barnet, Enfield and Haringey Mental Health Trust, London, United Kingdom; 2 Camden and Islington Foundation Trust, London, United Kingdom

## Abstract

**Aims:**

1. To evaluate clinicians’ experiences of the newly implemented remote ASD assessment process (due to COVID-19), including the long-term sustainability and potential standardisation of this approach; 2. To establish areas for improvement in this process and make further recommendations.

**Methods:**

Members of the Neurodevelopmental MDT completed an online survey, whereby feedback was collected regarding the use of the Child Observation of Social Communication (COSC), which had been adapted for online use from the standardised Autism Diagnostic Observation (ADOS) Schedule by a senior Psychologist[.Participants also responded to questions on other assessment domains, including the Developmental, Dimensional and Diagnostic interview, feedback and formulation meetings. Questions included their comfort with performing the assessment, theirs views on the quality of care provided and any difficulties they faced. Survey data were collected on two occasions: between November and December 2020 and between July and August 2021.

**Results:**

**Positive Experiences**

63% of respondents in November-December 2020 reported that COSC was a good alternative whilst standardised ADOS was unavailable. This increased to 100% in July-August 2021. Quality of care delivered by COSC was rated to be the same as ADOS in 70% of participants November-December 2020; 25% felt quality of care delivered by COSC was better than ADOS in July-August 2021. 73% of participants reported they would continue to use the remote assessment in the November-December 2020 survey. This increased to 88% in July-August 2021. 33% of the clinicians were very comfortable with administering the COSC in July-August 2021, 56% were somewhat comfortable.

**Negative Experiences**

27% of the clinicians reported being somewhat uncomfortable with administering the COSC assessment in November-December 2020; 11% remained somewhat uncomfortable in July-August 2021. 30% of the participants rated the quality of care delivered by COSC worse than ADOS in November-December 2020. 37.5% rated this to be worse in July-August 2021. 77% of the respondents had technical or organisational difficulties, which could result in missing non-verbal cues during the assessment.

**Conclusion:**

Clinicians’ experiences improved over time and with practice (34% had delivered over 10 COSC assessments in July-Aug 2021). A hybrid model may increase the quality of care of the approach, as well as careful selection of cases which would be suitable for an online assessment. There is scope for the continued use of the remote ASD pathway, taking into account patient and clinician preferences, however patient feedback will be necessary as a next step in this evaluation.

